# Effect of Filler Particle Size on the Mechanical and Acoustic Performance of Rigid Polyurethane Foam/Aluminosilicate Microsphere Composites

**DOI:** 10.3390/polym18151840

**Published:** 2026-07-27

**Authors:** Beata Zygmunt-Kowalska, Patrycja Zakrzewska, Artur Bukowczan, Renata Porębska, Andrzej Rybak, Aleksandra Chojak, Agnė Kairytė, Monika Kuźnia, Krzysztof Pielichowski

**Affiliations:** 1 Department of Heat Engineering and Environment Protection, Faculty of Metals Engineering and Industrial Computer Science, AGH University of Krakow, al. A. Mickiewicza 30, 30-059 Krakow, Poland; trestka@agh.edu.pl (P.Z.); achojak@agh.edu.pl (A.C.); kuznia@agh.edu.pl (M.K.); 2 Department of Chemistry and Technology of Polymers, Faculty of Chemical Engineering and Technology, Cracow University of Technology, Warszawska 24 St., 31-155 Krakow, Poland; artur.bukowczan@pk.edu.pl; 3 Interdisciplinary Centre for Circular Economy, Cracow University of Technology, Warszawska 24 St., 31-155 Krakow, Poland; 4 ABB Corporate Technology Center, Starowislna 13A St., 31-038 Krakow, Poland; renata.porebska@pl.abb.com (R.P.); andrzej.rybak@pl.abb.com (A.R.); 5 Laboratory of Thermal Insulating Materials and Acoustics, Institute of Building Materials, Faculty of Civil Engineering, Vilnius Gediminas Technical University, Linkmenų St. 28, 08217 Vilnius, Lithuania; agne.kairyte@vilniustech.lt

**Keywords:** microspheres, cellular structure, compressive strength, sound absorption coefficient, brittleness, rigid polyurethane foam

## Abstract

Rigid polyurethane foams (RPUFs) are widely used as lightweight thermal insulation materials. Their properties can be improved by incorporating inorganic fillers. However, the effect of filler particle size has not been sufficiently investigated. This study examines the influence of aluminosilicate microsphere diameter (80, 150, 300, and 500 μm) on the properties of RPUFs. Foams containing 10 wt.% microspheres (M) were prepared by the free-rise method. Their cellular structure, apparent density, mechanical, acoustic, thermal, and thermomechanical properties were evaluated. The addition of microspheres reduced the average cell diameter from 184 ± 29 μm for PU_0 to 147–174 μm, depending on microsphere size, and increased the apparent density from 31.3 to approximately 37 kg·m^−3^. The compressive strength decreased from 186 ± 6 kPa for PU_0 to 159 ± 2 kPa for PU_500M, whereas the tensile strength increased from 257 ± 14 kPa for PU_0 to 323 ± 14 kPa for PU_500M. The highest average sound absorption coefficient (0.14) was obtained for PU_300M, representing a 75% improvement over PU_0 (0.08). The composites also showed improved thermal stability and storage modulus. Among the investigated composites, PU_300M exhibited the most balanced combination of mechanical and acoustic properties.

## 1. Introduction

Rigid polyurethane foams (RPUFs) possess distinctive properties, including low thermal conductivity, chemical inertness, durability, and the capacity to adapt their properties to specific requirements [1]. RPUFs are synthesized via the polyaddition reaction between polyols and diisocyanates. During the foaming process, blowing agents are responsible for generating the characteristic cellular structure, while catalysts and surfactants control the reaction and stabilize the foam. The addition of other substances, including flame retardants and fillers, is a common practice to modify the properties of the resulting materials [2]. These properties make RPUFs suitable for a wide range of applications, including the construction, automotive, and furniture industries. The extensive range of applications for foams, as well as other polyurethanes (PUs), can be attributed to the rapid growth of the global polyurethane market, which is projected to reach an estimated value of approximately USD 120 billion by 2030 [3]. Conventional RPUFs are produced from petrochemical feedstocks, and their production has a considerable environmental impact [4]. Growing environmental awareness has stimulated research on more sustainable polyurethane materials. Current research focuses on replacing petrochemical polyols with bio-based alternatives derived from vegetable oils or biomass [5,6], developing sustainable diisocyanates, improving recyclability, and designing isocyanate-free polyurethane systems [7,8]. At the same time, fillers derived from renewable resources or industrial by-products are increasingly used to modify the properties of polyurethane foams while promoting more efficient resource utilization.

Numerous studies have demonstrated that fillers can significantly improve the mechanical and functional performance of RPUFs [9,10,11,12,13,14]. Clay fillers have been used to reduce gas diffusion through the foam cell walls, thereby improving the long-term thermal insulation performance of polyurethane foams [15]. Cement-based fillers reinforce the cellular structure of polyurethane foams, leading to higher stiffness and yield strength under compressive loading [16]. Silica nanoparticles enhance the thermal stability and chemical stability of polyurethane foams, enabling their use under harsh chemical and environmental conditions [17]. Recently, bio-based silica nanoparticles obtained from agricultural waste have also been reported to improve the thermal insulation and compressive strength of RPUFs while enabling the valorization of biomass waste [18]. However, few publications address the effect of filler particle size on the final properties of RPUFs, despite this aspect being of key importance. The evaluation of the effect of filler particle size is of significance, as it affects the homogeneity of the mixture, the distribution of the filler in the polyurethane matrix, and thus the strength and thermal insulation properties of the material. Large filler particles or their agglomeration may result in an inhomogeneous filler distribution and structural defects, which adversely affect the mechanical performance of polyurethane foams. In contrast, smaller particles are generally dispersed more uniformly within the polymer matrix, promoting a more homogeneous cellular structure and improved mechanical stability [19]. Lorusso et al. investigated the effect of titanium dioxide (TiO_2_) nanoparticles and halloysite nanotubes (HNTs) on rigid polyurethane foams. They reported that the incorporation of 4–8 wt.% HNTs reduced the thermal conductivity, which was attributed to the elongated shape of the nanotubes that increased the number of nucleation sites, resulting in a finer cellular structure and improved thermal insulation. In contrast, the highest mechanical performance was achieved for foams containing 6 wt.% TiO_2_ nanoparticles, which reinforced the cell walls [20]. Conversely, larger filler particles have been shown to exert a detrimental effect on the mechanical properties of the material. This assertion is corroborated by the findings of Thirumal et al. [21] who incorporated fillers, including silica, calcium carbonate, and glass powder, into rigid polyurethane foam at concentrations ranging from 5 wt.% to 50 wt.%. The experimental observations indicated that as the filler concentration increased, the material’s mechanical properties exhibited a decline.

The mechanical properties of RPUF composites depend primarily on three factors: material’s microstructure, filler type and concentration. Notably, the mechanical properties are closely related to the density of the foam, as confirmed by Goods et al. [22]. Furthermore, the mechanical properties of foams are influenced by the mechanical properties of the particles that are incorporated into the RPUFs system. The reinforcing effect depends on the manner in which the filler particles are incorporated into the foam matrix. In their study, Saint-Michel et al. [19] evaluated the effect of filler particle size on the mechanical properties of foams. In their study, they utilized filler calcium carbonate sizes of 1 and 30 um. The study demonstrated that the filler size exerts a substantial influence on the mechanical properties of the foams. In addition to experimental studies, the authors of the publication carried out modelling of the mechanical properties. The comparison of mechanical characterization results with different modeling approaches showed that considering the filler size relative to the foam wall thickness is crucial for accurately describing its viscoelastic properties and yield stress. Two modeling assumptions were made: one treating the composite foam as a filler dispersion in the foam and the other as a void dispersion in the filled polymer. The first model better describes foams with large calcium carbonate particles, while the second is more suitable for foams with small particles. It was determined that the size of the filler, to the wall thickness, is a salient issue when preparing the model. In the case of porous materials, sound absorption can only occur in a structure with open pores. It is caused by viscous and thermal losses present in the interconnected channels formed in the material. Rigid polyurethane foams exhibit a predominantly closed-cell structure, which limits their sound absorption performance. The incorporation of suitable fillers may modify the cellular structure and enhance acoustic energy dissipation, thereby improving the sound absorption properties of the foams [23,24].

Previous studies have demonstrated that inorganic fillers can improve the mechanical, thermal, and acoustic properties of rigid polyurethane foams. However, most studies have focused on the type or content of the filler. The influence of filler particle size has received limited attention. In particular, the effect of aluminosilicate microsphere diameter on the mechanical, thermal, and acoustic performance of RPUFs has not been systematically investigated. Aluminosilicate microspheres are lightweight hollow particles recovered from coal fly ash. They combine low density, low thermal conductivity, and high crushing strength while enabling the valorization of an industrial by-product [25]. Therefore, this study investigates the effect of microsphere diameter on the cellular structure, mechanical, thermal, thermomechanical, and acoustic properties of RPUF composites. Unlike previous studies, the filler content was kept constant to evaluate only the effect of microsphere diameter. The findings provide new insight into the role of filler particle size and may facilitate the design of lightweight insulating materials for construction and transportation applications.

## 2. Materials and Methods

### 2.1. Materials

**Figure 1:**
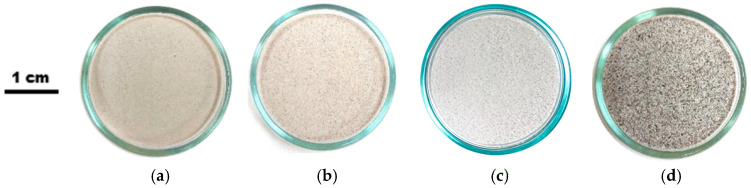
Photographs of microsphere fractions: 80 µm (**a**), 150 µm (**b**), 300 µm (**c**), and 500 µm (**d**).

The filler used in the study was aluminosilicate microspheres obtained from coal combustion fly ash (EKO-EXPORT S.A., Bielsko-Biała, Poland). The study utilized commercially classified aluminosilicate microsphere fractions with nominal average diameters of approximately 80, 150, 300, and 500 µm (Figure 1).

Rigid polyurethane foams (RPUFs) were prepared using the commercial two-component polyurethane system EKOPRODUR PM4032 (PCC Prodex Sp. z o.o., Brzeg Dolny, Poland). Component A (EKOPRODUR PM4032) was a formulated polyol blend, whereas Component B (EKOPRODUR B) was a mixture of aromatic polyisocyanates, mainly polymeric diphenylmethane diisocyanate (pMDI). The manufacturer’s recommended weight ratio of Component A to Component B was 100:110.

### 2.2. Preparation of RPUF Composites

Rigid polyurethane foam composites containing 10 wt.% aluminosilicate microspheres were prepared by the free-rise method. Aluminosilicate microspheres were initially dispersed in Component A using a mechanical stirrer. Subsequently, Component B was added according to the manufacturer’s recommended weight ratio (100:110, Component A:Component B), and the mixture was stirred at 2000 rpm for 10 s. The reaction mixture was immediately poured into an open mold and allowed to freely expand under ambient laboratory conditions. The foams were left to cure for 48 h at room temperature before being cut into specimens for further characterization. A neat polyurethane foam without microspheres was prepared following the same procedure and served as the reference sample.

The filler content was selected based on our previous investigations, in which 10 wt.% of microsphere fillers was found to provide beneficial mechanical and thermal performance while maintaining an intact cellular structure. In contrast, higher filler contents resulted in noticeable disruption of the foam morphology [26]. The sample designations and their descriptions are presented in Table 1.

### 2.3. Characterization

The aluminosilicate microspheres were characterized by scanning electron microscopy (SEM) and Fourier-transform infrared spectroscopy (FTIR). To verify the particle size of the commercially classified microsphere fractions, SEM images were acquired using a JEOL JSM5410 scanning electron microscope (JEOL, Peabody, MA, USA). FTIR spectra of the microspheres were recorded using a Thermo Scientific Nicolet iS5 spectrometer (Thermo Scientific, Waltham, MA, USA) equipped with an iD7 ATR accessory. The spectra were collected in the wavenumber range of 4000–400 cm^−1^ with a spectral resolution of 4 cm^−1^. As the FTIR spectra of all microsphere fractions were nearly identical, only the spectrum of the 80 μm microspheres is presented as a representative example. Primarily, the density of rigid foams was assessed in accordance with the ISO 845 standard [27]. The microstructural examination of modified RPUFs was conducted using JEOL JSM5410 SEM. Before the analysis, the foams were sputter-coated with a thin gold layer under vacuum using a QUORUM Q150R ES (Quorum Technologies Ltd., Lewes, UK). The horizontal cell diameter and cell-wall thickness were determined from the SEM images using ImageJ software (version 1.53k; National Institutes of Health, Bethesda, MD, USA). The horizontal cell diameter was measured perpendicular to the foam rise direction, whereas the cell-wall thickness was determined as the distance between the boundaries of adjacent cells. The closed-cell content was measured by ISO 4590, method 2 [28].

The compressive and tensile strength tests were conducted following ISO 29469 [29] and EN 1607 [30] standards using the H10KS machine (Hounsfield, UK). The reported compressive strength corresponds to the compressive stress at 10% deformation. The specific compressive and tensile strength values were calculated by dividing the measured strength by the apparent density of the corresponding foam sample.

Friability was determined using the oak box method by ASTM C421–08 [31].

The sound absorption coefficient was measured using a Brüel & Kjær 4207T impedance tube equipped with two ¼″ B&K 4187 microphones, a B&K 2716-C amplifier, a B&K 3160 analyzer, and PULSE LabShop v16 software (Brüel & Kjær, Nærum, Denmark). The measurements were conducted in the frequency range of 50–6300 Hz. Two characteristics were analyzed: the sound absorption coefficient in 1/3-octave bands and the average sound absorption coefficient. For each material variant, two samples (100 mm and 29 mm in diameter) with a thickness of approximately 3 cm were prepared. A constant thickness was maintained to isolate the effect of microsphere addition on the acoustic performance of the foam.

Thermogravimetric (TG) analysis was performed using a TG 209 F1 Libra thermogravimetric analyzer (Netzsch, Selb, Germany). Approximately 2.5 mg of each sample was heated in an open α-Al_2_O_3_ crucible under a nitrogen atmosphere at a heating rate of 10 K min^−1^. The residue content was determined at 650 °C. The evolved gases were analyzed using an Alpha II FTIR spectrometer (Bruker Optics, Ettlingen, Germany) coupled to the TG analyzer. Representative FTIR spectra recorded at 330 °C were used for further analysis.

Dynamic mechanical analysis (DMA) was performed using a DMA 242 C analyzer (Netzsch, Selb, Germany) in compression mode under a nitrogen atmosphere (50 mL min^−1^). Cubic specimens were cooled with liquid nitrogen and heated from −150 to 200 °C at a rate of 3 K min^−1^. The measurements were carried out at a frequency of 1 Hz, using an oscillation amplitude of 40 µm, a proportional factor (PF) of 1.1, and a maximum dynamic force of 5 N.

## 3. Results and Discussion

### 3.1. Characterization of Microspheres as a Filler

**Figure 2:**
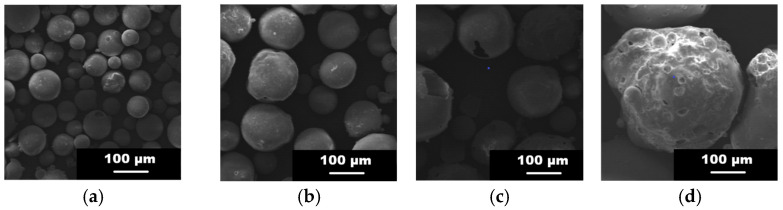
SEM images of microsphere fractions with nominal particle sizes of 80 µm (**a**), 150 µm (**b**), 300 µm (**c**), and 500 µm (**d**).

Figure 2 presents SEM micrographs of the microsphere fillers, illustrating their morphology and surface characteristics.

The SEM observations confirmed that the microspheres were consistent with the nominal average diameters provided by the manufacturer. The observed morphology, characterized by predominantly spherical particles with smooth outer surfaces, is consistent with previous reports on aluminosilicate microspheres derived from coal fly ash [32]. No significant particle deformation or structural defects were observed. These results indicate good filler quality and suggest that the differences in foam properties are primarily related to the microsphere size.

**Figure 3:**
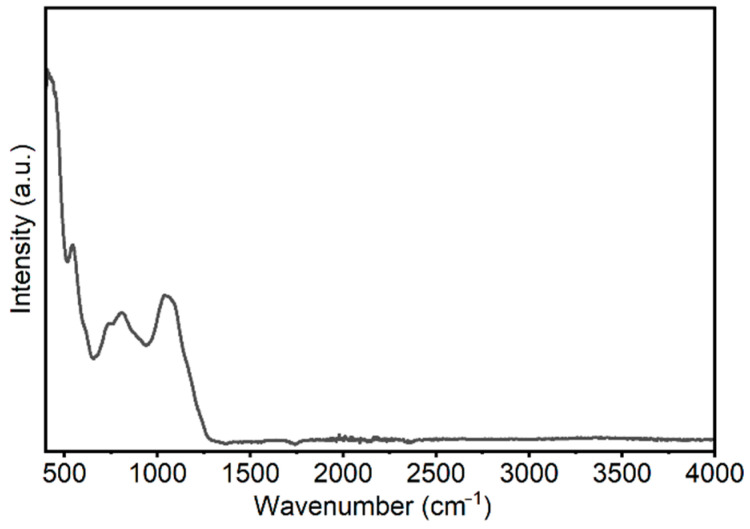
Representative FTIR spectrum of the 80 μm aluminosilicate microspheres.

Figure 3 presents the representative FTIR spectrum of the 80 μm aluminosilicate microspheres. The spectra of the remaining microsphere fractions were nearly identical.

The spectrum exhibits characteristic absorption bands of aluminosilicate materials. The broad band centered at approximately 1040 cm^−1^ is assigned to the asymmetric stretching vibrations of Si-O-Si and Al-O-Si bonds. The bands at approximately 790 and 460 cm^−1^ correspond to the symmetric stretching and bending vibrations of Si–O bonds, respectively. These results confirm the aluminosilicate nature of the microspheres.

### 3.2. Effect of Microspheres on the Properties of Polyurethane Foams

#### 3.2.1. Cellular Morphology and Apparent Density

**Figure 4:**
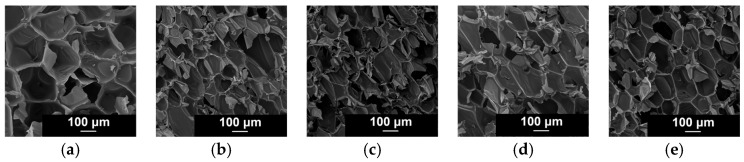
SEM images of PU_M samples: PU_0 (**a**), PU_80M (**b**), PU_150M (**c**), PU_300M (**d**) and PU_500M (**e**).

Figure 4 and Table 2 present the cellular morphology of PU_M samples. In addition, apparent density, a key parameter commonly used to characterize polyurethane foams was investigated and summarized also in Table 2.

The addition of microspheres modified the cellular structure of the polyurethane foams, resulting in a reduction in cell diameter and wall thickness compared with the reference foam. The average cell diameter decreased by approximately 20%, indicating that the microspheres acted as heterogeneous nucleation sites during foaming, promoting the formation of a greater number of cells while suppressing their growth and coalescence. Similar nucleating effects of inorganic fillers in rigid polyurethane foams have been reported previously, where solid particles promoted bubble nucleation and suppressed cell coalescence [20,33]. Consequently, the modified foams exhibited a finer and more homogeneous cellular structure. Simultaneously, the apparent density increased by nearly 15%, indicating that the presence of microspheres increased the viscosity of the reacting system, thereby restricting bubble expansion and producing a more compact cellular structure [34]. This interpretation is supported by the increase in closed-cell content from 88% for the reference foam to 94–97% for the modified foams. The higher fraction of closed cells may be attributed not only to the reduced coalescence of adjacent bubbles but also to the stabilizing effect of the rigid microspheres, which locally reinforced the thin polymer membranes and reduced their susceptibility to rupture during foam expansion [35]. As the microsphere diameter increased, the cell walls became slightly thicker, suggesting a greater local influence of larger particles on polymer distribution within the cell walls.

#### 3.2.2. Mechanical and Acoustic Properties

**Figure 5:**
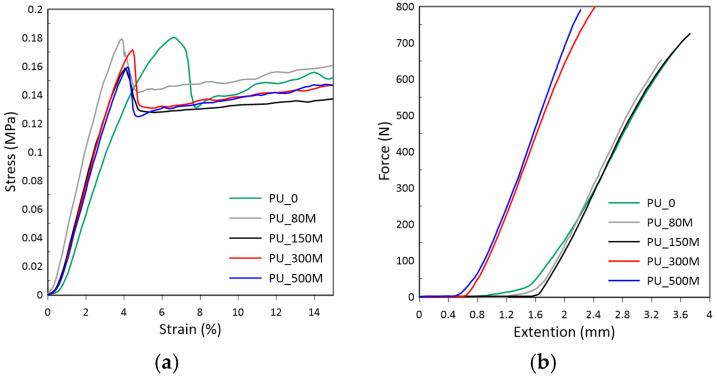
Compression test results of PU_M samples: stress–strain curves (**a**) and force–extension curves (**b**).

The mechanical and acoustic properties of the PU_M samples are summarized in Table 3. The compressive strength, tensile strength, friability, and average sound absorption coefficient were evaluated to assess the influence of microsphere diameter on the overall performance of the polyurethane foams. Representative stress–strain and force–extension curves obtained during the compression and tensile tests are presented in Figure 5.

The results summarized in Table 3 indicate that the addition of microspheres affected both the mechanical and acoustic performance of the polyurethane foams. Compressive strength gradually decreased with increasing microsphere diameter, whereas tensile strength exhibited the opposite trend. The highest tensile strength was obtained for PU_500M (323 ± 14 kPa), while the PU_0 exhibited the highest compressive strength (186 ± 6 kPa). The specific mechanical properties indicate that the effect of microsphere diameter cannot be explained solely by changes in apparent density. Instead, the modified cellular morphology plays the dominant role in determining the mechanical performance of the foams. The mechanical properties of the foams are closely related to changes in cellular morphology presented in Table 2. The incorporation of microspheres reduced the average cell diameter and significantly decreased the cell-wall thickness compared with the reference foam. At the same time, the apparent density and closed-cell content increased. The reduced cell-wall thickness explains the gradual reduction in compressive strength, as they are more susceptible to local buckling and collapse under compressive loading, which is consistent with the established relationship between foam cellular morphology and mechanical performance [36]. In contrast, the higher apparent density and more homogeneous cellular structure contributed to the increase in tensile strength, particularly for PU_300M and PU_500M. The friability of all modified foams was higher than that of the reference material, indicating that microsphere incorporation increased the susceptibility of the foam surface to mechanical damage. Furthermore, the refined cellular structure and higher closed-cell content likely contributed to the improved sound absorption coefficient, with the best acoustic performance observed for PU_300M. Curves presented in Figure 5 confirm these trends. The lower compressive strength of the modified foams can be attributed to changes in the cellular structure caused by the incorporation of microspheres, resulting in reduced load-bearing capacity under compression. In contrast, the improved tensile strength may be related to the modified cellular morphology observed after microsphere incorporation. This behavior is consistent with previous studies demonstrating that cellular morphology strongly influences the mechanical performance of rigid polyurethane foams [33].

**Figure 6:**
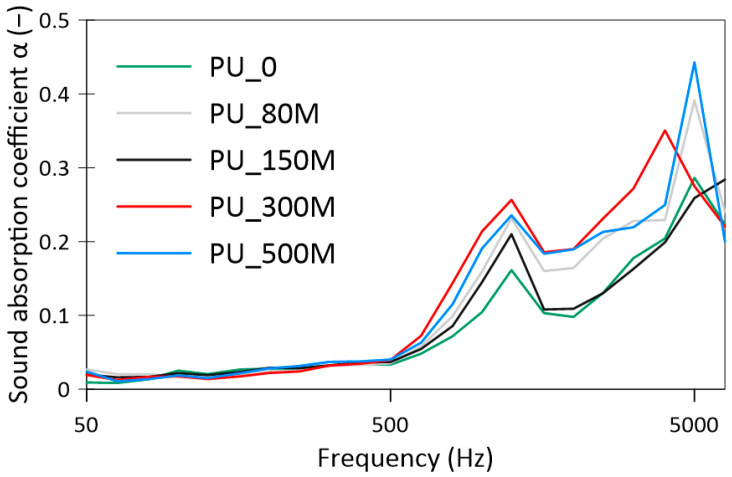
Sound absorption coefficient of PU_M samples.

The sound absorption results are summarized in Table 3 and illustrated in Figure 6. For all samples, the sound absorption coefficient increased with frequency and exhibited resonance peaks. The incorporation of microspheres improved the acoustic performance of the polyurethane foams, although the extent of improvement depended on the microsphere diameter. The highest average sound absorption coefficient was obtained for PU_300M, representing an approximately 75% increase compared with the reference foam. The improved acoustic performance can be directly related to the modified cellular morphology [37]. The reduction in cell diameter, together with the increase in apparent density and closed-cell content produced a finer and more compact cellular structure. Such a morphology increases the tortuosity of the sound propagation path, promoting greater viscous losses during sound-wave propagation through the foam [38]. Furthermore, microspheres embedded within the cell walls may increase viscoelastic damping by locally restricting polymer chain mobility. During acoustic excitation, deformation of the cell walls generates interfacial shear stresses at the polymer–microsphere interface, facilitating the conversion of acoustic energy into heat [39,40]. In addition, microscopic interfacial heterogeneities or partial debonding around the microspheres, although not reflected in the measured closed-cell content, may create locally acoustically active pathways that further enhance energy dissipation. Despite the increase in closed-cell content, the sound absorption improved, indicating that the enhanced acoustic performance was governed primarily by increased tortuosity, viscoelastic damping and interfacial energy dissipation introduced by the microspheres rather than by airflow through open cells. Interestingly, PU_300M exhibited higher sound absorption than PU_500M, suggesting that acoustic performance is governed not only by microsphere size but also by an optimal balance between cellular morphology, wall stiffness, and interfacial interactions.

Although the improvements observed were significant relative to the reference foam, the absolute values remained too low for practical sound-absorbing applications. Further enhancement may be achieved by increasing the filler content and optimizing the microsphere size.

#### 3.2.3. Thermal and Thermomechanical Properties

**Figure 7:**
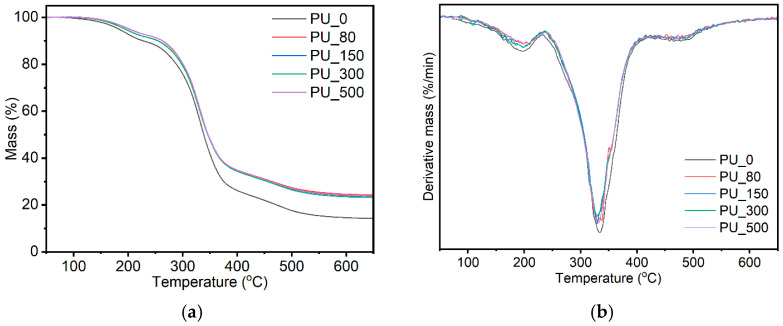
TG (**a**) and DTG (**b**) curves of PU_M samples.

The thermal stability of the PU_M samples was evaluated by TGA. The TG and DTG curves are shown in Figure 7, while the characteristic degradation parameters are summarized in Table 4.

Figure 7a shows that all samples exhibited the typical two-stage degradation profile of polyurethane foams. The first stage of degradation, associated with the breakdown of the hard urethane segments, occurred at temperatures of approximately 180–250 °C, while the second stage, associated with the degradation of the soft segments, was observed at temperatures of approximately 330–340 °C [41,42,43]. These stages are confirmed by two peaks visible on the DTG curves (Figure 7b).

The incorporation of microspheres improved the initial thermal stability of the composites, as evidenced by higher T_5_% and T_10_% values compared to the PU_0. The greatest improvement was observed for PU_80M, where the T_5_% and T_10_% values increased by approximately 19 °C and 35 °C, respectively. These results indicate that the incorporation of microspheres delayed the onset of thermal degradation, which can be attributed to the barrier effect of the inorganic filler [21]. In many composite systems, such energy absorption is often followed by a steeper TG profile caused by the subsequent release of energy; however, in this case, this effect was not observed, indicating that the microspheres delay the onset of degradation without accelerating the subsequent stages of decomposition [44].

The residue content increased from 14.3% for PU_0 to 23.2–24.4% in the PU_M samples; it confirms the 10% microspheres load as the inorganic filler particles do not undergo thermal decomposition in the temperature range used during this study.

**Figure 8:**
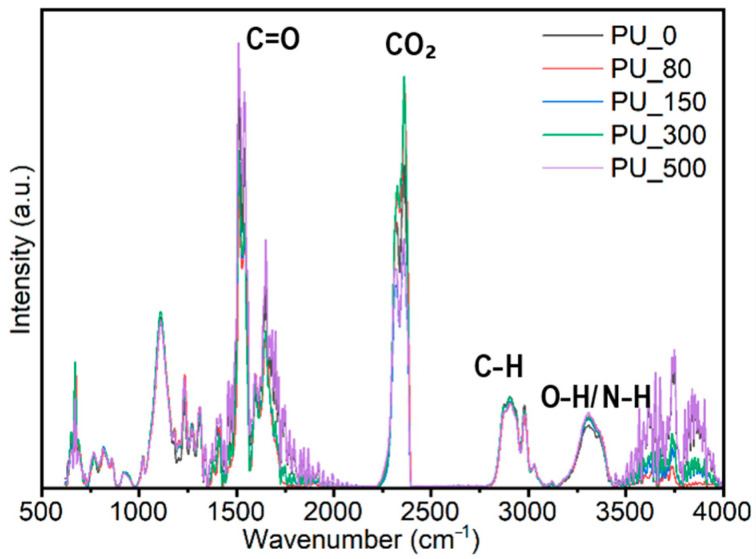
TG-FTIR recorded at 330 °C of PU_M samples.

The gaseous products formed during the thermal decomposition of PU_0 and PU_M samples were analyzed using the TG-FTIR method at a temperature of 330 °C, corresponding to the main decomposition stage (Figure 8). All samples exhibited similar FTIR spectra, indicating the formation of the same volatile decomposition products. In all samples, characteristic absorption bands were observed that are attributed to CO_2_ (approx. 2350 cm^−1^), compounds containing a carbonyl group (1700–1800 cm^−1^), hydrocarbons (2800–3000 cm^−1^), and O–H/N–H stretching vibrations (3200–3700 cm^−1^) [45].

The only differences observed were minor variations in the intensity of individual bands; however, no new absorption bands or significant shifts in peak positions were observed. This indicates that the introduction of microspheres did not alter the degradation mechanism or the chemical nature of the volatile products formed during decomposition [46]. These results confirm that the changes observed in the TGA results are primarily due to physical effects, such as heat absorption and delayed heat transfer by the microspheres, rather than changes in the chemical degradation pathway of the polyurethane matrix.

**Figure 9:**
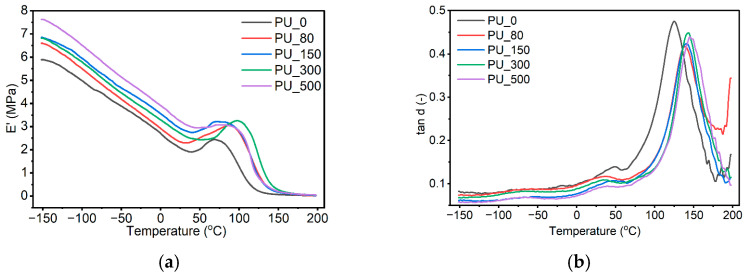
Storage modulus (**a**) and loss tangent (**b**) at 1 Hz of PU_M samples.

Figure 9 shows the temperature dependence of the storage modulus (E′) and loss tangent (tan δ).

At low temperatures (around −150 °C), all samples exhibited high values of the storage modulus, indicating a glassy state in which the mobility of the polymer chains is severely restricted. As the temperature increased, the E′ value gradually decreased due to increased segment mobility and the appearance of a glass-transition region. PU_0 exhibited a more pronounced and earlier decrease in stiffness compared to the PU_M samples, indicating lower thermal resistance. In contrast, the modified samples maintained higher modulus values over a wider temperature range, which demonstrates the reinforcing effect of the microspheres and an improvement in the thermomechanical stability of the polyurethane matrix [47].

In the temperature range from approximately 80 to 120 °C, a transition region associated with segmental relaxation and glass-transition processes was observed. For microsphere-modified foams, this region shifted slightly toward higher temperatures, suggesting a restriction of polymer chain dynamics resulting from stronger interactions between the filler and the matrix.

As shown in Figure 9b, a distinct increase in the tan δ value was observed in the 80–120 °C temperature range, which corresponds to the glass-transition temperature (Tg) and the associated molecular relaxations. The reference foam exhibited a sharper and more intense peak at slightly lower temperatures, while the modified foams exhibited broader peaks shifted toward higher temperatures. This behavior indicates reduced chain mobility and increased structural heterogeneity caused by the presence of microspheres. Furthermore, the broadening and shift in the tan δ maximum suggest changes in the segmental dynamics of the polyurethane matrix and a more complex internal structure of the composite foams, which is consistent with previous reports for polyurethane composites containing microsphere fillers [48].

## 4. Conclusions

This study demonstrated that the diameter of aluminosilicate microspheres is a key factor governing the morphology and performance of rigid polyurethane foam composites.

The incorporation of microspheres modified the cellular structure by reducing the average cell diameter from 184 ± 29 μm for the reference foam to 147–174 μm for the modified foams, while simultaneously increasing the apparent density from 31.3 to approximately 37 kg·m^−3^ and the closed-cell content from 88% to 96–97%. The changes in cellular morphology were reflected in the mechanical properties. Increasing the microsphere diameter resulted in a gradual decrease in compressive strength from 186 ± 6 kPa to 159 ± 2 kPa, whereas the tensile strength increased from 257 ± 14 kPa for the reference foam to 323 ± 14 kPa for PU_500M. Although the incorporation of microspheres increased foam friability, larger microspheres reduced this effect compared with the smaller fractions. The highest average sound absorption coefficient (0.14) was obtained for PU_300M, corresponding to an approximately 75% improvement compared with the reference foam (0.08). In addition, the incorporation of microspheres increased the initial thermal stability and storage modulus without altering the thermal degradation mechanism of the polyurethane matrix.

The results demonstrate that controlling filler particle size is an effective approach to tailoring the multifunctional performance of rigid polyurethane foams. Among the investigated fractions, microspheres with a diameter of 300 μm provided the most advantageous balance between mechanical, acoustic, and thermomechanical properties, making them a promising filler for advanced lightweight polyurethane composites.

## Figures and Tables

**Table 1:** Sample identification.

Sample	Description
PU_0	Neat foam
PU_80M	Foam with 10 wt.% microspheres (average diameter: ~80 µm)
PU_150M	Foam with 10 wt.% microspheres (average diameter: ~150 µm)
PU_300M	Foam with 10 wt.% microspheres (average diameter: ~300 µm)
PU_500M	Foam with 10 wt.% microspheres (average diameter: ~500 µm)

**Table 2:** Apparent density and morphology of PU_M samples.

Sample	Horizontal Cell Diameter (µm)	Wall Thickness (µm)	Closed-Cell Content (%)	Apparent Density (kg·m^–3^)
PU_0	184 ± 29	36 ± 8	88 ± 1	31.3 ± 0.1
PU_80M	147 ± 35	15 ± 6	94 ± 0	36.1 ± 0.4
PU_150M	165 ± 31	17 ± 4	96 ± 1	37 ± 0.8
PU_300M	174 ± 35	20 ± 5	97 ± 2	37 ± 0.9
PU_500M	169 ± 40	23 ± 2	96 ± 2	36.5 ± 0.6

**Table 3:** Mechanical and acoustic properties of PU_M samples.

Sample	Compressive Strength (kPa)	Specific Compressive Strength (kPa·m^3^·kg^−1^)	Tensile Strength (kPa)	Specific Tensile Strength (kPa·m^3^·kg^−1^)	Friability (%)	Average Sound Absorption Coefficient (-)
PU_0	186 ± 6	5.94	257 ± 14	8.21	3.0 ± 0.7	0.08
PU_80M	178 ± 3	4.93	277 ± 19	7.67	6.2 ± 0.5	0.10
PU_150M	169 ± 9	4.57	293 ± 9	7.92	5.5 ± 0.9	0.09
PU_300M	168 ± 4	4.54	303 ± 22	8.19	5.3 ± 0.5	0.14
PU_500M	159 ± 2	4.36	323 ± 14	8.85	4.7 ± 0.9	0.12

**Table 4:** Thermal stability parameters of PU_M samples.

Sample	T_5%_ (°C)	T_10%_ (°C)	T_50%_ (°C)	DTG_MAX1_ (°C)	DTG_MAX2_ (°C)	Residue at 650 °C (%)
PU_0	183.5	228.5	339.0	197.1	334.0	14.3
PU_80M	202.5	263.5	347.0	198.1	329.7	24.4
PU_150M	196.0	255.5	346.5	198.2	329.1	23.7
PU_300M	195.5	256.0	346.0	198.3	329.1	23.2
PU_500M	203.5	264.5	346.5	198.8	329.8	23.8

## Data Availability

The original contributions presented in this study are included in the article. Further inquiries can be directed to the corresponding author(s).
